# Photocatalytic Degradation of Inherent Pharmaceutical Concentration Levels in Real Hospital WWTP Effluents Using g-C_3_N_4_ Catalyst on CPC Pilot Scale Reactor

**DOI:** 10.3390/molecules28031170

**Published:** 2023-01-25

**Authors:** Ilaeira Rapti, Theodora Kourkouta, Evrydiki-Maria Malisova, Triantafyllos Albanis, Ioannis Konstantinou

**Affiliations:** 1 Laboratory of Industrial Chemistry, Department of Chemistry, University of Ioannina, 45110 Ioannina, Greece; 2 Institute of Environment and Sustainable Development, University Research Center of Ioannina (URCI), 45110 Ioannina, Greece

**Keywords:** pharmaceuticals, hospital wastewaters, solar photocatalysis, g-C_3_N_4_, pilot plant, solar photocatalysis

## Abstract

The objective of this work was to evaluate the efficiency of a solar photocatalytic process using g-C_3_N_4_ as photocatalyst on the degradation of pharmaceutical compounds detected in hospital wastewater treatment plant secondary effluents. A compound parabolic collector pilot plant, established in the secondary effluent stream of the Ioannina city hospital wastewater treatment plant, was used for the photocatalytic experiments. The analysis of the samples before and after the photocatalytic treatment was accomplished using solid phase extraction (SPE), followed by UHPLC-LTQ/Orbitrap HRMS. Initial effluent characterization revealed the presence of ten pharmaceutical compounds. Among these, amisulpride, O-desmethyl venlafaxine, venlafaxine and carbamazepine were detected in all experiments. Initial concentrations ranged from 73 ng L^−1^ for citalopram to 2924.53 ng L^−1^ for O-desmethyl venlafaxine. The evolution of BOD_5_ and COD values were determined before and after the photocatalytic treatment. All detected pharmaceuticals were removed in percentages higher than 54% at an optimum catalyst loading ranging between 200 and 300 mg L^−1^. The potential of the catalyst to be reused without any treatment for two consecutive cycles was studied, showing a significant efficiency decrease.

## 1. Introduction

Water is a natural resource essential to life, however, a large part of the population faces tough shortages and/or poor quality of water. Today, water systems are frequently polluted by a range of chemicals that are a direct result of industrial development and anthropogenic activities, including heavy metals, synthetic dyes, and agrochemicals (i.e., pesticides and fertilizers). Over the last two decades, a wide range of emerging organic pollutants originating from a variety of sources, such as pharmaceutical and personal care compounds, flame retardants and plasticizers, have frequently been detected in water systems. In particular, the presence of pharmaceutical pollutants is becoming an increasing environmental problem, and developing strategies for their successful removal is an essential goal for current research.

Pharmaceuticals are substances or mixtures of substances with therapeutic or preventive properties used to treat human diseases. However, they are also an important category of micropollutants resulting from point or diffuse pollution. Only partial removal of pharmaceutical pollutants has been demonstrated by conventional wastewater treatment strategies, and as a result, municipal and/or hospital WWTPs have emerged as major sources of their release into the environment. Pharmaceutical compounds reach aquatic media at concentrations ranging from ng L^−1^ to μg L^−1^. Research has shown that even at low concentrations, pharmaceuticals can induce adverse effects on both the ecosystem (reduction of biodiversity, extinction of aquatic organisms, etc.) and human health [1,2,3,4,5]. 

For the efficient removal and degradation of pharmaceutical compounds from wastewaters, and the prevention of their release to ground and surface waters, innovative technologies, such as advanced oxidation processes (AOPs)—which have been suggested as tertiary treatment in effluent wastewater—have been developed. AOPs comprise different processes of generating reactive oxygen species (ROS), mainly hydroxyl radicals, and are considered to be promising methods for the remediation of contaminated wastewaters containing non-biodegradable organic pollutants. Heterogeneous photocatalysis, which is based upon the efficient production of ROS in aqueous media, is able to attain complete removal of various pharmaceutical pollutants [6,7,8].

During the last decade, a wide variety of photocatalysts have been developed. Of these, graphitic carbon nitride (g-C_3_N_4_) combines several advantages, such as having a mild band gap (2.7 eV), a good response to visible light (up to 460 nm), a simple preparation methodology, high chemical stability and non-toxicity, and being low cost, which make it suitable for a broad range of photocatalytic applications [9,10]. Nevertheless, there are only a small number of published research studies in relation to hospital wastewaters. Zhong et al. [11] studied the photocatalytic degradation of carbamazepine under simulated solar irradiation using g-C_3_N_4_ in spiked real wastewater effluent collected from a full-scale WWTP located in Brisbane, Queensland, Australia, and they reported 100% degradation of carbamazepine after 15 min. In addition, Kane et al. [12] studied the photodegradation of carbamazepine using g-C_3_N_4_ under UV light irradiation. Gao et al. [13] studied the removal efficiency of amoxicillin in real hospital wastewater and in a wastewater treatment plant, using a composite g-C_3_N_4_-based photocatalytic material, Ag/TiO_2_/M-g-C_3_N_4_. After 60 min of the photocatalytic process, a 71% and 49% removal of amoxicillin was observed in the two sample types, respectively.

Among various reactor types, the compound parabolic concentrator (CPC) has been extensively suggested as one of the best solar reactor configurations for pilot and full-scale photochemical applications. CPC reactors consist of cylindrical glass reactors mounted on a combined reflecting profile. They provide the best conditions for low-concentration solar systems. CPC reactors can function during cloudy days due to the design of the mirror, which allows almost all radiation (direct and diffuse) to reach the CPC [14,15,16,17].

In this work, the application of heterogeneous photocatalysis using various loadings of graphitic carbon nitride (g-C_3_N_4_) in a pilot solar CPC reactor was employed to study the degradation of inherent pharmaceutical compounds present in hospital wastewater secondary effluents. The reusability of the photocatalyst was evaluated for two consecutive cycles under the same operating conditions. In addition, the physicochemical parameters of the effluent were monitored before and after the photocatalytic treatment. This is one of the first reports on the application of g-C_3_N_4_ under real-world conditions for the treatment of hospital wastewaters, since previous studies have dealt with either laboratory scale reactors or distilled and/or synthetic wastewaters.

## 2. Results

### 2.1. Degradation of Pharmaceuticals in Hospital WWTP Effluent

Table 1 presents the physicochemical parameters of the hospital secondary wastewater samples (min, max, standard deviation (S.D.) and median values), which represented the typical range values of such effluents [15,18,19]. The removal rates for total phenolic content at the end of the photocatalytic treatment with 100 and 300 mg L^−1^ g-C_3_N_4_ were 11% and 15.2%, respectively, while the absorbances at 254 nm decreased by 19.0% and 19.6%, respectively.

Ten pharmaceutical compounds were measured at detectable inherent concentration levels in the WWTP secondary effluents. The photocatalytic degradation of these pharmaceutical compounds was studied under solar irradiation using g-C_3_N_4_ as photocatalyst at varying concentrations (100, 200 and 300 mg L^−1^). Amisulpride, venlafaxine, O-desmethyl venlafaxine (the major metabolite of venlafaxine), and carbamazepine were found in all experiments. Valsartan and citalopram were detected in the experiments where the g-C_3_N_4_ concentration was 100 and 300 mg L^−1^. Quetiapine was detected only once, in the experiment with 100 mg L^−1^ g-C_3_N_4_; while trimethoprim, mirtazapine and atenolol were detected only in the experiment with 300 mg L^−1^ g-C_3_N_4_. The initial concentration levels of the pharmaceuticals ranged from 73 ng L^−1^ for citalopram to 2924.53 ng L^−1^ for O-desmethyl venlafaxine, representing the low to medium concentration ranges previously recorded for these pharmaceuticals in secondary hospital or municipal wastewaters [15,20]. 

**Figure 1:**
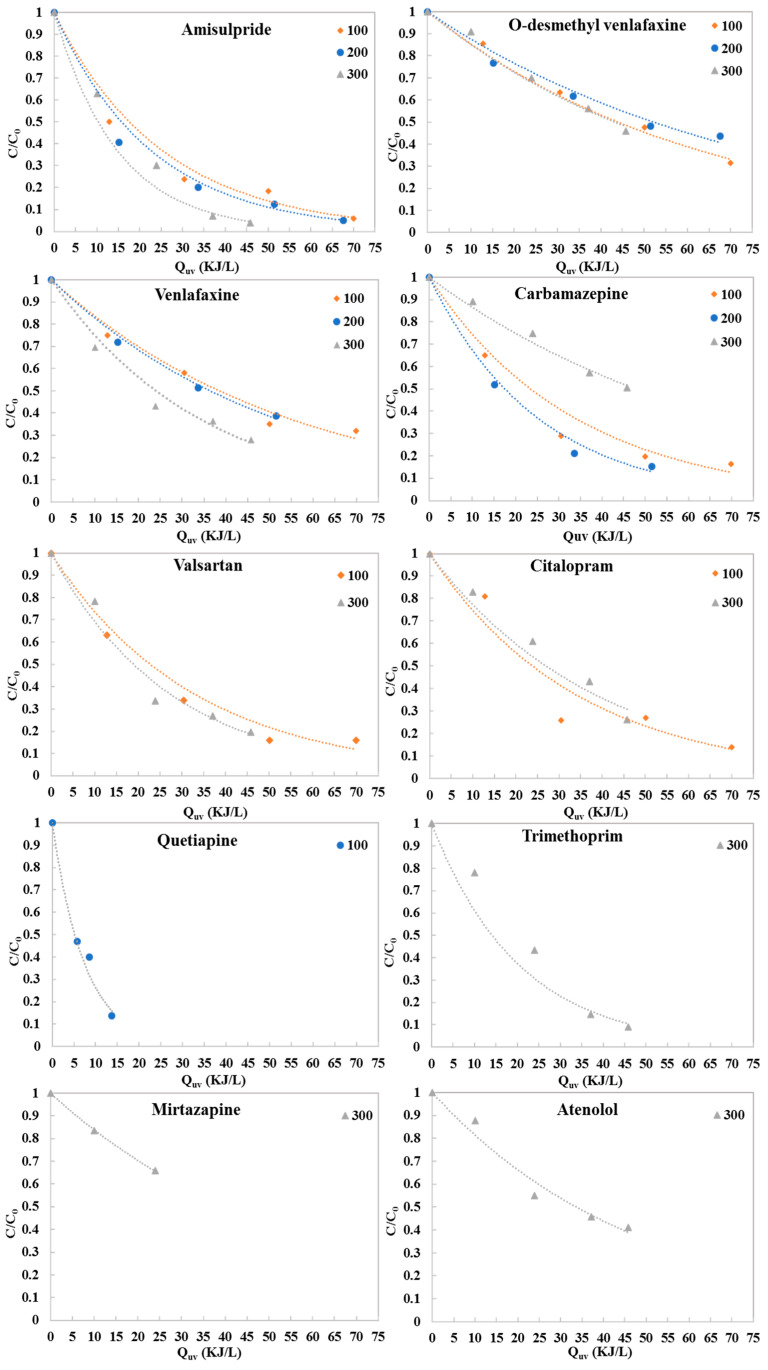
Photocatalytic degradation of pharmaceutical compounds detected in hospital WWTP secondary effluents using different g-C_3_N_4_ concentrations in a pilot-scale PCP photoreactor as a function of accumulative UV energy per liter of effluent.

**Figure 2:**
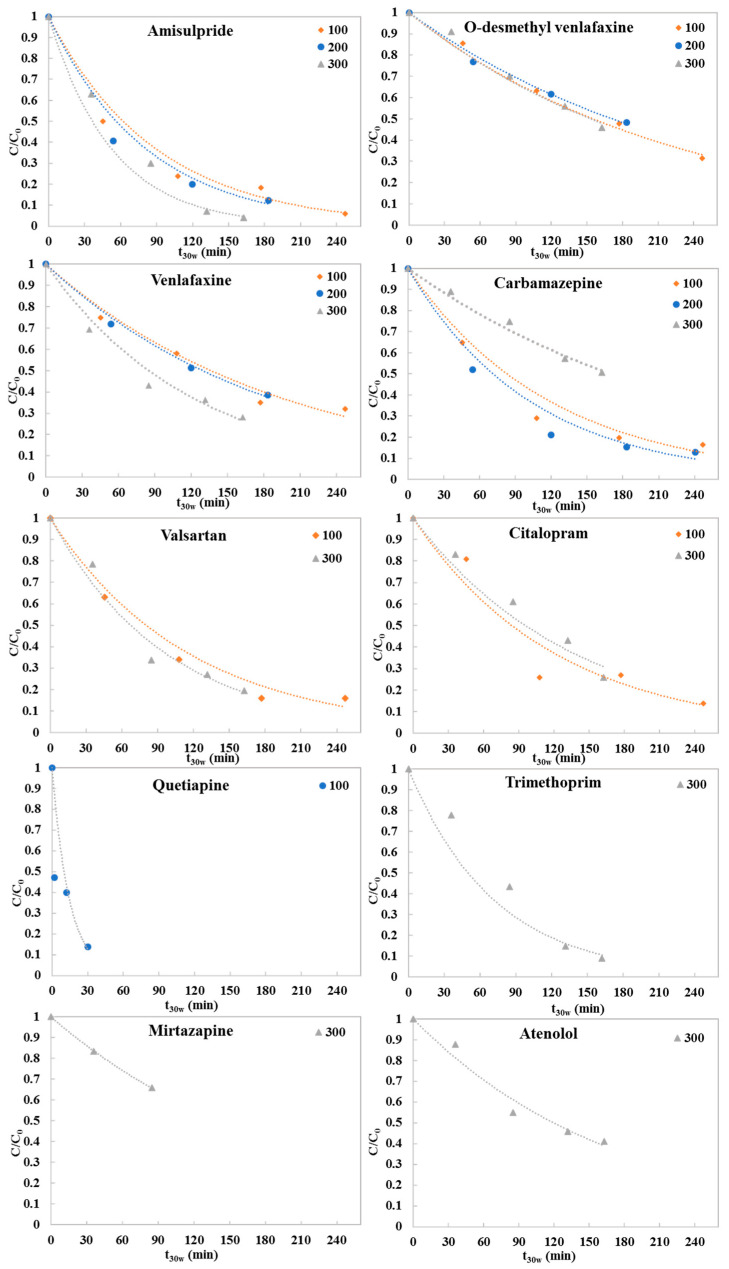
Photocatalytic degradation of pharmaceutical compounds detected in hospital WWTP secondary effluents using different g-C_3_N_4_ concentrations in a pilot-scale PCP photoreactor as a function of normalized illumination time t_(30w)_.

Most of the studied pharmaceuticals were effectively degraded by g-C_3_N_4_ photocatalysis. At the end of the photocatalytic process using 300 mg L^−1^ g-C_3_N_4_, after 4 h of natural solar irradiation, the recorded removal percentages ranged between 34% for mirtazapine and 96% for amisulpride. Their degradation profiles fitted well with pseudo-first order kinetics, always having R^2^ values above 0.965. The photocatalytic degradation kinetics of the pharmaceuticals under different concentrations of g-C_3_N_4_, as a function of accumulative UV energy per liter of effluent, are presented in Figure 1, while the corresponding kinetics as a function of normalized illumination time t_30w_ are presented in Figure 2. The photocatalytic degradation rate constants (L kJ^−1^), the correlation coefficients (R^2^) and the percent degradation (%R) of the pharmaceuticals are summarized in Table 2 and Table 3.

The photocatalytic degradation experiments showed that the rate constants and removal percentages of amisulpride, O-desmethyl venlafaxine, venlafaxine and valsartan increased as the photocatalyst loading was increased. Specifically, using 300 mg L^−1^ g-C_3_N_4_, the degradation percentages were above 72% for amisulpride, venlafaxine and valsartan, when the accumulative energy was 45.78 kJ L^−1^ and the t_30w_ was 162 min. According to the kinetic constants, the photocatalytic efficiency when using 300 mg L^−1^ g-C_3_N_4_ followed the trend amisulpride > trimethoprim > valsartan > venlafaxine > citalopram > atenolol > mirtazapine > O-desmethyl venlafaxine > carbamazepine. In contrast, for carbamazepine and citalopram, the presence of a higher concentration of catalyst reduced its activity. The degradation of citalopram was 86% (accumulative energy 69.93 kJ L^−1^) with 100 mg L^−1^ g-C_3_N_4_; while for carbamazepine it was 87% (accumulative energy 67.61 kJ L^−1^) with 200 mg L^−1^ g-C_3_N_4_. A degradation of 86% was achieved for quetiapine at 13.76 kJ L^−1^ accumulated energy and t_30w_ 30 min using 100 mg L^−1^ g-C_3_N_4_; while 87% of the carbamazepine was degraded at 67.61 kJ L^−1^ accumulated energy and t_30w_ 240 min using 200 mg L^−1^ of the catalyst.

Previously published studies on the application of g-C_3_N_4_ for the degradation of pharmaceuticals include only lab-scale experiments. As a case study, Dou et al. [21] studied the photocatalytic degradation of the two typical β-lactam antibiotics, amoxicillin and cefotaxime, present in hospital wastewater secondary effluents using g-C_3_N_4,_ reporting that after 60 min of the photocatalytic process, a 60% and 90% removal of amoxicillin and cefotaxime, respectively, was achieved. Furthermore, Moreira et al. [22] studied the photocatalytic degradation of eight pharmaceutical compounds (carbamazepine, clopidogrel, diclofenac, atenolol, bezafibrate, tramadol, venlafaxine and fluoxetine) found in the biologically treated effluents of an urban wastewater treatment plant located in the northern region of Portugal using exfoliated g-C_3_N_4_. At the end of the photocatalytic process, an almost complete removal of all compounds was observed.

**Figure 3:**
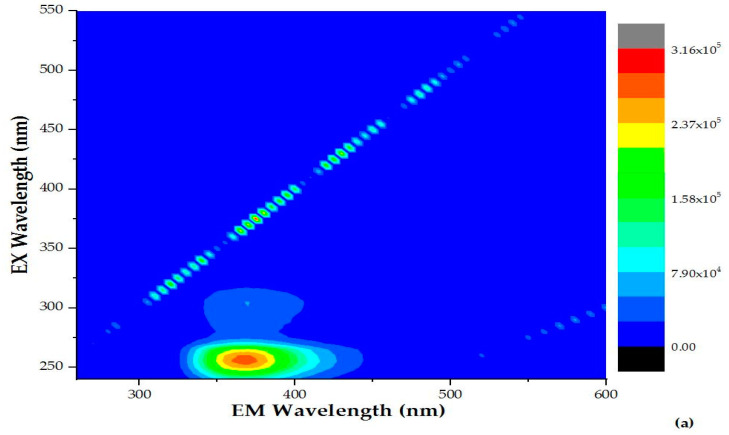

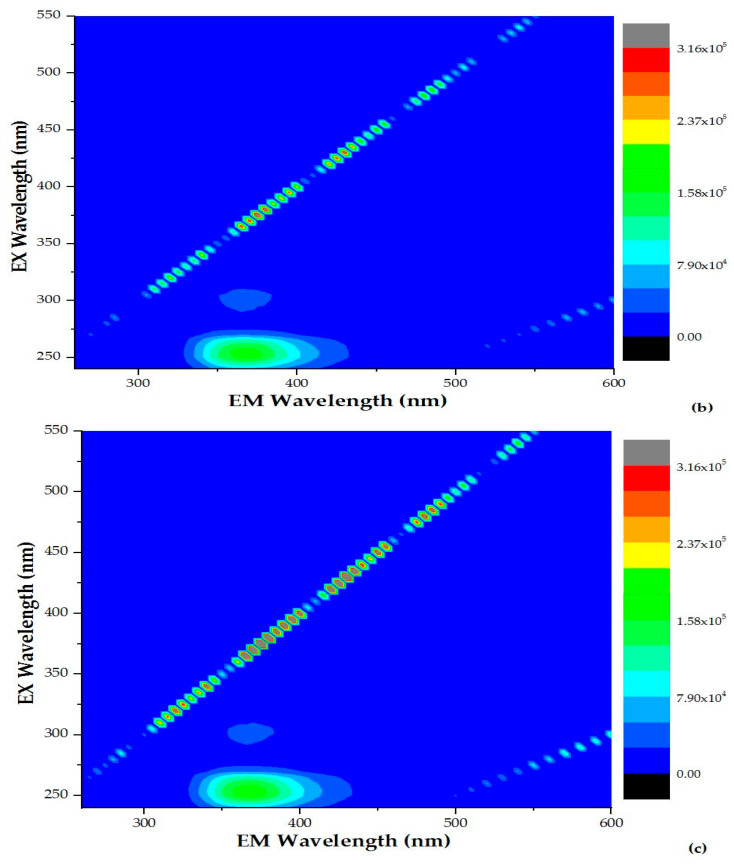
Evolution of fluorescence EEM matrix; (**a**) t = 0 min, (**b**) t = 120 min and (**c**) t = 240 min of photocatalytic treatment.

Recent studies have employed the measurement of fluorescence peaks from excitation–emission matrices (EEMs) as a monitoring tool for the photocatalytic oxidation of dissolved organic matter (DOM) [23]. Figure 3 represents the evolution of fluorescence EEM matrix after 0, 120 and 240 min of photocatalytic treatment. Samples of secondary effluent (t = 0 min) presented a fluorescence peak at Ex/Em of 260/365 nm (Peak T region), indicating the dominant presence of tryptophan-like or protein-like and some phenolic compounds [24]. Such peaks are often found in aquatic systems subject to anthropogenic input and/or in sewage samples [24,25]. After photocatalytic treatments for 240 min (45.78 kJ L^−1^) of solar irradiation, this peak intensity showed a decrease of approximately 40 %.

**Figure 4:**
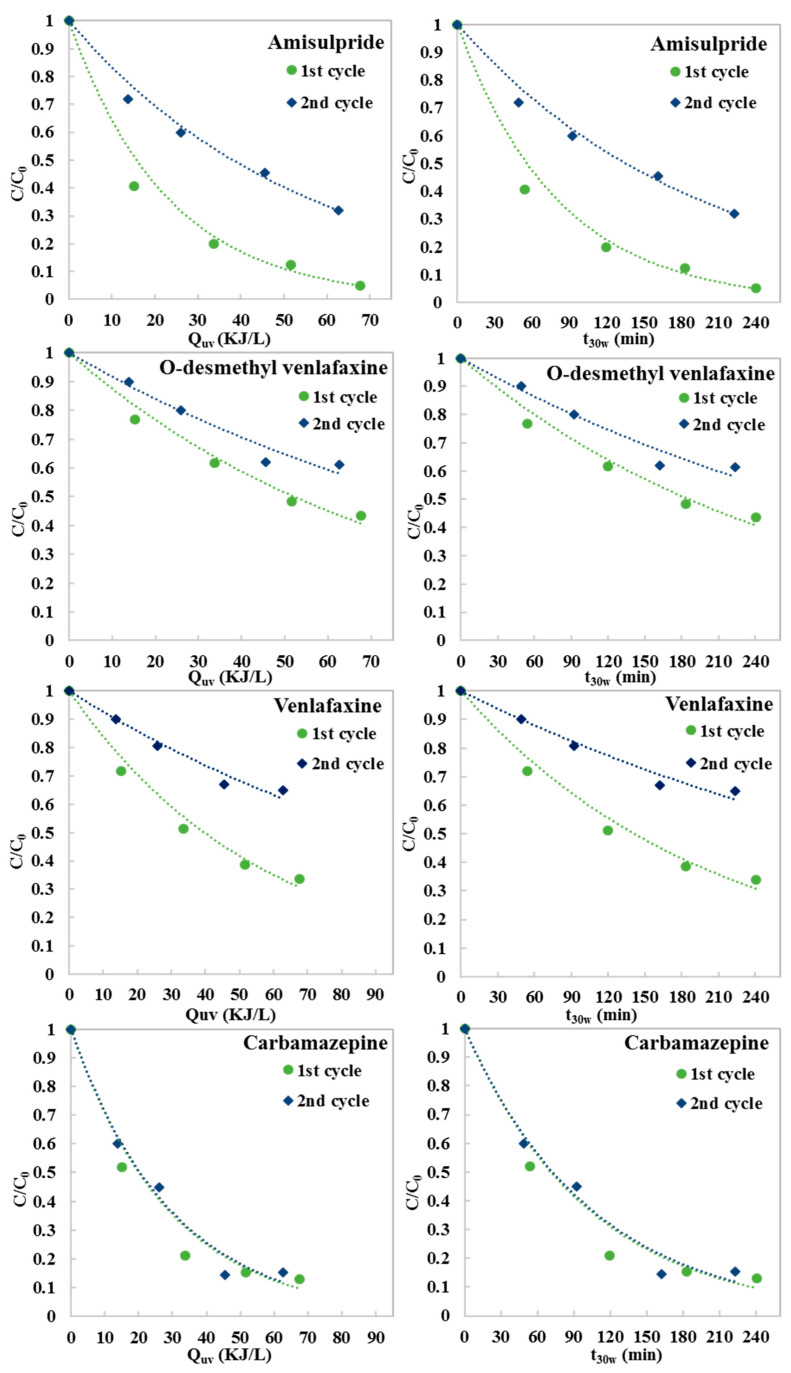
Photocatalytic degradation of PhaCs over two consecutive catalytic cycles (200 mg L^−1^ g-C_3_N_4_) as a function of accumulative UV energy per liter of effluent and normalized illumination time (t_30w_).

In the experiments dealing with the reusability of the g-C_3_N_4_ photocatalyst, four pharmaceuticals were detected; specifically, amisulpride, venlafaxine, O-desmethyl venlafaxine and carbamazepine were found at concentrations ranging between 796.65 ng L^−1^ for carbamazepine and 2504.39 ng L^−1^ for O-desmethyl venlafaxine. The photocatalytic degradation rate constants (L kJ^−1^), the correlation coefficients (R^2^) and the percentage degradation (%R) of the pharmaceuticals have been summarized in Table 2. Figure 4 shows the degradation of these pharmaceutical compounds over two consecutive catalytic cycles using 200 mg L^−1^ g-C_3_N_4_, as a function of accumulative UV energy per liter of effluent (Q_uv_) and normalized illumination time (t_30w_).

In the first cycle, the pharmaceutical compound degradation ranged between 56% and 95% for O-desmethyl venlafaxine and amisulpride, respectively. In the second cycle, a significant loss of photocatalytic efficiency was observed, with the pharmaceutical compound removal ranging from 35% for venlafaxine to 84% for carbamazepine. The degradation rate constants in the second catalytic cycle decreased by 59% for amisulpride, 43.7% for O-desmethyl venlafaxine, 57.9% for venlafaxine and 15% for carbamazepine. Taking into account the higher initial COD concentration, as well as the fact that the catalyst was reused without any pretreatment, the observed decrease can be attributed to an increased scavenging activity of the organic matter, and the accumulation of inorganic and organic compounds onto the catalyst surface, as also described elsewhere. In addition, some minor losses of catalyst can be considered owing to partial sedimentation of the finer catalyst particles. Heterogeneous photocatalyst suspension systems should be followed by effective systems for the recovery of catalysts when expanding the technology to higher technology readiness levels.

### 2.2. Organic Load Evolution

Table 4 shows the data for the five-day biological oxygen demand (BOD_5_) (mg L^−1^), the chemical oxygen demand (COD) (mg L^−1^) and the BOD_5_/COD ratio measured in secondary effluent before and after treatment using different catalyst loadings. This biodegradability assessment of wastewater is of practical significance as it is a useful metric when optimizing wastewater treatment processes to achieve maximum removal efficiency. According to literature data, a BOD_5_/COD ratio of 0.4–0.6 indicates good biodegradability of the wastewater [26]. The BOD_5_/COD ratio increased in all experiments after the photocatalytic treatment. 

## 3. Materials and Methods

### 3.1. Reagents and Chemicals

All reagents used in the experiments were analytical grade and used as received without further purification. Bupropion hydrochloride (BUP) and mirtazapine (MTZ) were obtained from LGC (Wesel, Germany). Amisulpride (AMS), amitriptyline (AMT), fluoxetine hydrochloride (FLX), olanzapine (OLN), paroxetine (PRX) and venlafaxine hydrochloride (VNX) were purchased from TCI (Zwijndrecht, Belgium). Bezafibrate (BZF), budesonide (BUD), caffeine (CAF), carbamazepine (CBZ), citalopram (CIT), clofibric acid (CA), clozapine (CZP), diclofenac (DCF), fenofibrate (FNB), fluvoxamine (FXM), gemfibrozil (GMF), haloperidole (HAL), norfluoxetine hydrochloride (NFX), O-desmethyl venlafaxine (ODV), quetiapine (QTP), risperidone (RIS), salicylic acid (SA), sertraline hydrochloride (STR), simvastatin (SIM), sulfadiazine (SDZ), sulfamethoxazole (SMX), sulfapyridine (SPY), sulfathiazole (STZ), tolfenamic acid (TA) and trimethoprim (vetranal) (TMP) were purchased from Sigma-Aldrich (Darmstadt, Germany). N-desmethyl olanzapine (DMO) and N-desmethyl sertraline hydrochloride (DMS) were purchased from Santa Cruz Biotechnology (Santa Cruz, CA, USA). The structures and main physicochemical properties of pharamceutical compounds are presented in Table S1. Amitriptyline-d6 hydrochloride, carbamazepine-d_10_, fluoxetine-d_5_ hydrochloride, haloperidol-d_4_ and olanzapine-d_3_ were supplied by A2S (Saint Jean d’Illac, France). Individual stock solutions of each compound, as well as isotopically labeled internal standard solutions, were prepared in methanol and stored at −20 °C. LC-MS grade methanol and water, and Na_2_EDTA were purchased from Fisher Scientific (Leicestershire, UK). Folin–Ciocalteu’s phenol reagent, Bis-Tris and formic acid (purity: 98–100%) were obtained from Merck KGaA (Darmstadt, Germany). Sodium carbonate and p-hydroxy benzoic acid were supplied by Sigma Aldrich (St. Louis, MO, USA). Boric acid was obtained from Supelco (Bellefonte, PA, USA). Oasis HLB (200 mg, 6 cm^3^) cartridges used for solid phase extraction were bought from Waters Corporation (Milford, MA, U.S.A.). Graphitic carbon nitride (g-C_3_N_4_) (Specific Surface Area: 35 m^2^g^−1^, particle size of 25 nm, E_g_ = 2.82 eV) synthesis and characterization used as photocatalyst are described in a previous publication from our group [9].

### 3.2. Photocatalytic Experiments

**Figure 5:**
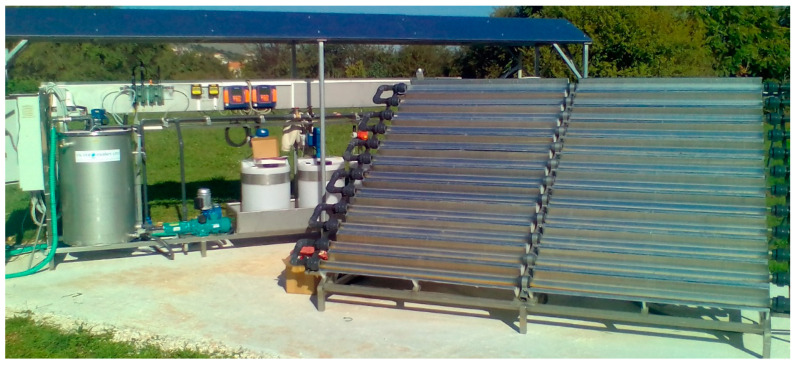
Photograph of CPC photocatalytic pilot plant established in the secondary effluent of WWTP facility of University Hospital, Ioannina city.

Photocatalytic experiments were carried out under sunlight at the University Hospital WWTP of Ioannina (Northwestern Greece), where a compound parabolic collector (CPC) reactor pilot plant is established. The hospital has a capacity of 800 beds. The waste first enters into the screening tank and then into the equilibration tank. Biological treatment is then carried out in the activated sludge aeration tank, followed by sedimentation. The hydraulic retention time (HRT) is 6 h, while the solid retention time (SRT) is 1.5 days. The photocatalytic pilot plant (Figure 5) consists of parabolic compound collectors exposed to sunlight; a reservoir tank of working volume 300 L, with an air blower and mechanical stirrer; a recirculation pump; and connecting tubing. The collector consists of 24 borosilicate glass tubes (dimensions: 55 mm × 1.5 m, wall thickness 1.8 mm). The total volume of the reactor is 85 L, while the irradiated surface is 12 m^2^. The CPC photoreactor tube module is connected to a recirculation tank and a centrifugal pump. For online measurements of solar intensity, temperature, flow rate, pH and dissolved oxygen (DO), several sensors are installed. The reactor is operated in continuous flow recirculation mode.

The experiments were started by adding secondary effluent to the equilibration tank of the CPC unit. The first wastewater sample was taken for analysis after 15 min of homogenization using the mechanical stirrer and air blower. To avoid photoreaction, the collectors were covered with a radiation-resistant textile. Next, the appropriate amount of g-C_3_N_4_ was added, and the system stirred for 1 h in continuous flow recirculation mode to achieve a homogeneous suspension. In order to evaluate the adsorption of the contaminants onto the surface of the catalyst, another sample was taken. Then, the reactor was exposed to sunlight and samples were collected at predefined times. The samples were filtered (0.45 μm, HVLP filter) and kept at 4 °C until their extraction on the same day. The sensors also recorded online UV solar radiation, temperature and pH.

The removal of the pharmaceutical compounds was plotted as the C/C_0_ against the accumulative UV energy during exposure time per unit of treated water volume (Q_uv_, kJ L^−1^) and the normalized illumination time (t_30w_, min). Accumulative UV energy during exposure time per unit of treated water volume was calculated using Equation (1), while normalized illumination time was determined using Equation (2):

(1) $$Q_{\mathrm{uv},n+1}=Q_{\mathrm{UV}}\;+{\Delta t}_{n}\;\cdot \;{\bar{\mathrm{UV}}}_{G,n+1}\;\cdot \;\frac{A_{i}}{V_{T}}$$

(2) $$t_{30W,n+1}=t_{30W}\;+{\Delta t}_{n}\;\cdot \;\frac{{\bar{\mathrm{UV}}}_{G,n+1}}{30}\;\cdot \;\frac{V_{i}}{V_{T}}\;{\Delta t}_{n}=t_{n+1}-\;t_{n}$$

where t_30W_ (min) is illumination time, Q_UV_ (kJ L^−1^) is the accumulated UV energy per unit of volume needed to reach a particular degradation level for each sample and UV_G,n + 1_ (W m^−2^) is the average solar UV radiation (λ < 400 nm) measured between t_n + 1_ and t_n_. A_i_ is the irradiated surface and t_30w_ is the “normalized illumination time”. V_T_ is the total volume of the secondary effluent loaded into the pilot plant and V_i_ is the total irradiated volume [3,17].

Reuse of the catalyst in the pilot scale CPC was tested using 200 mg L^−1^ g-C_3_N_4_. The catalyst was not washed between the catalytic cycles, and was left to settle in a sedimentation tank. BOD_5_, COD, total phenolic content and absorbance at 254 nm were measured before and after the photocatalytic treatment for each cycle.

### 3.3. Determination of Physicochemical Parameters

Five-day biochemical oxygen demand (BOD_5_) was measured by means of a WTW OxiTop OC 110 system and a WTW TS 606-G/2-i thermostat cabinet (WTW, Weilheim, Germany). The chemical oxygen demand (COD) was determined using a WTW Thermoreactor 3200 and a WTW pHotoFlex portable photometer, following the corresponding set of test (WTW, Weilheim, Germany). Inorganic ions were determined using an ion chromatograph (Shimadzu CDD-6A) equipped with a column (Shimadzu Shim-pack IC-A3 150 × 4.00 mm, 5 μm), a column oven (CTO-10A VP) and a conductivity detector (Shimadzu CBM-20A). The mobile phase consisted of 50 mmol boric acid, 3.2 mmol/L BisTris and 8 mmol/L p-hydroxy benzoic acid, flowing at a rate of 1.1 mL min^−1^. The temperature was set at 40 °C. The volume injected onto the chromatograph was 50 μL. The total phenolic content of the samples was determined using the Folin–Ciocalteau (FC) colorimetric assay. A 5 mL portion of the sample was mixed with 250 μL of FC reagent. After 2 min, 750 μL of Na_2_CO_3_ solution was added. After 1 h of standing in dark, the absorbance was measured at a wavelength of 750 nm using a UV–Vis spectrophotometer (Jasco-V630, Tokyo, Japan). Absorbance at 254 nm was determined using a UV–Vis spectrophotometer (Jasco-V630, Tokyo, Japan) in order to indirectly evaluate the aromatic compound content. Fluorescence emission–excitation matrices (EEMs) of the secondary and photocatalytically treated wastewaters were measured using a Fluoromax+ (Horiba) luminescence spectrometer, by means of scanning the emission wavelengths (Em) from 260 to 600 nm at 1 nm-increments and stepping through the excitation wavelengths (Ex) from 240 to 550 nm at 5 nm intervals. Excitation and emission slits were both adjusted to 1 nm. 

### 3.4. Extraction of Wastewater Samples

After adjustment to approximately pH 7 and addition of 2 mL of Na_2_EDTA, samples were submitted to solid phase extraction (SPE). Oasis HLB cartridges (200 mg, 6 cm^3^) were first conditioned with 5 mL LC-MS grade methanol and 5 mL LC-MS grade water. A volume of 100 mL of sample was loaded onto the conditioned SPE cartridges under vacuum, at a flow rate of 5 mL min^−1^. Then, 5 mL of LC-MS grade methanol was added to wash the cartridges, which were then dried under vacuum for 15 min. Elution was performed using 5 mL of LC-MS grade methanol twice, at a flow rate of 1 mL min^−1^. The eluent was evaporated to dryness under a gentle stream of nitrogen by means of a Techne Dri-Block heater, model DB-3D. Rehydration of the samples was conducted using 500 μL of methanol:water 20:80 (*v*/*v*) containing 0.1% formic acid. The samples were stored at −20 °C until analysis.

### 3.5. LTQ-FT Orbitrap Instrument Operational Parameters

The samples were analyzed using a UHPLC Accela LC system connected to a hybrid LTQ-FT Orbitrap XL 2.5.5 SP1 mass spectrometer equipped with an electrospray ionization source (ESI) (Thermo Fisher Scientific, Bremen, Germany), as reported in our previous work. Chromatographic separation of the target analytes was performed using a reverse phase Hypersil Gold C18 (Thermo Fisher Scientific, San Jose, CA, USA) analytical column (100  ×  2.1 mm, 1.9 μm particle size) maintained at 27 °C. Full scans in positive and negative ionization mode were acquired for identification and quantification of the pharmaceutical compounds. The mobile phase for positive ionization (PI) consisted of solvent (A): LC-MS grade water with 0.1% formic acid, and solvent (B): LC-MS grade methanol with 0.1% formic acid. The elution gradient started at 95% A and remained for 1 min, progressed to 30% at 3 min, and then to 0% at 6 min, and finally returned to 95% A after 3 min, with re-equilibration of the column set at 1 min. In negative ionization (NI) mode, the mobile phase was a mixture of solvent (A): LC-MS grade water, and solvent (B): LC-MS grade methanol. The elution gradient started with 90% A for 0.5 min, progressed to 30% at 2 min, reaching 10% at 3 min, then it was decreased to 5% at 3.9 min, decreased again to 0% at 4.5 min, and remained at this for 0.5 min. After 1 min, it was returned to 90% A with re-equilibration of the column set at 2 min. Total run time was 8 min. The flow rate was 0.4 mL/min and the injection volume was 20 μL in both cases. For the data-dependent acquisition (full MS/dd-MS^2^), collision-induced dissociation (CID) was performed. All data were processed using Thermo Xcalibur 2.1 software (Thermo Electron, San Jose, CA, USA) [20].

## 4. Conclusions

The results of the present study showed that g-C_3_N_4_-assisted photocatalysis at pilot plant scale is a promising process for the degradation of pharmaceuticals detected in real hospital wastewater effluent. Degradation of pharmaceutical compounds followed pseudo-first order kinetics. All detected pharmaceuticals were removed at percentages higher than 54% at optimum catalyst loadings ranging between 200 and 300 mg L^−1^. Maximum degradation percentages at the end of the photocatalytic process reached 96% and 91% for amisulpride and trimethoprim, respectively—values which were achieved at 45.78 kJ L^−1^ accumulated energy and t_30w_ 162 min with 300 mg L^−1^ g-C_3_N_4_—while 87% degradation of carbamazepine was observed using 200 mg L^−1^ g-C_3_N_4_ (accumulative energy 67.61 kJ L^−1^). Other effluent quality parameters, such as phenolic content, UV-absorbance and biodegradability, were also ameliorated after photocatalytic treatment. With the exception of carbamazepine degradation, a significant reduction in the efficiency of the photocatalyst occurred after the second cycle, showing that the reusability of the photocatalyst remains one of the major challenges in slurry photocatalytic reactors.

## Figures and Tables

**Table 1:** Min, max, standard deviation (S.D.) and median values of physicochemical parameters measured in secondary effluent of hospital WWTP.

Physicochemical Parameters	Min	Max	S.D.	Median
pH	5.5	7.3	0.58	6.65
Temperature (°C)	12.2	25.7	3.8	21.8
Conductivity (μS cm^−1^)	1060	1959	300.9	1305
Total Dissolved solids (TDS, mg L^−1^)	308	1951	646.62	390
Turbidity (NTU)	4.1	16.5	3.5	10.4
BOD_5_ (mg L^−1^)	4.2	48.5	18.37	12.7
COD (mg L^−1^)	5	186	67.4	36.5
Abs_254_	0.16	0.31	0.03	0.25
Total phenols (mg L^−1^)	0.09	6.9	2.73	1.45
NO_3_^−^ (mg L^−1^)	1.89	50.9	19.2	26.4
PO_4_^3−^ (mg L^−1^)	2.96	50.6	12.64	10.04
Cl^−^ (mg L^−1^)	93.5	569.03	43.04	186.35
SO_4_^−^ (mg L^−1^)	1.57	45.7	8.1	12.7

**Table 2:** Kinetic parameters (first-order kinetic constants (k, L kJ^−1^), correlation coefficients (R^2^) and % removal (%R) for the corresponding accumulative energy (AE, kJ L^−1^) of pharmaceuticals after photocatalytic treatment using different catalyst loadings (C_g-C3N4_).

	Amisulpride			O-Desmethyl Venlafaxine			Venlafaxine			Carbamazepine		
C_g-C3N4_ (mg L^−1^)/(AE, kJ L^−1^)	k (L kJ^−1^)	R^2^	%R	k (L kJ^−1^)	R^2^	%R	k (L kJ^−1^)	R^2^	%R	k (L kJ^−1^)	R^2^	%R
100/(69.93)	0.039	0.9777	94	0.016	0.9947	69	0.018	0.9833	68	0.03	0.9747	83
200/(67.61) (1st cycle)	0.044	0.965	95	0.013	0.9861	56	0.019	0.9958	66	0.04	0.9927	87
300/(45.78)	0.067	0.9746	96	0.016	0.9867	54	0.029	0.9818	72	0.014	0.9889	49
200/(62.72) (2nd cycle)	0.018	0.9889	68	0.009	0.9648	39	0.008	0.9743	35	0.034	0.9851	84

**Table 3:** Kinetic parameters (first-order kinetic constants (k, L kJ^−1^), correlation coefficients (R^2^) and % removal (%R) for the corresponding accumulative energy (AE, kJ L^−1^) of pharmaceuticals after photocatalytic treatment using different catalyst loadings.

	g-C_3_N_4_ 100 (mg L^−1^)			g-C_3_N_4_ 300 (mg L^−1^)		
	k (L kJ^−1^)	R^2^	%R (AE)	k (L kJ^−1^)	R^2^	%R (AE)
Valsartan	0.03	0.9857	84 (69.93)	0.037	0.9721	84 (45.78)
Citalopram	0.029	0.9345	86 (69.93)	0.026	0.9742	74 (45.78)
Quetiapine	0.133	0.9858	86 (13.76)			
Trimethoprim				0.049	0.9517	91 (45.78)
Mirtazapine				0.018	0.9997	34 (23.93)
Atenolol				0.021	0.9716	59 (45.78)

**Table 4:** BOD_5_ (mg L^−1^), COD (mg L^−1^) and BOD_5_/COD ratio measured in secondary effluent of hospital WWTP before and after photocatalytic treatment using different catalyst loadings.

Parameter	100 mg L^−1^ g-C_3_N_4_		200 mg L^−1^ g-C_3_N_4_ (1st cycle)		200 mg L^−1^ g-C_3_N_4_ (2nd cycle)		300 mg L^−1^ g-C_3_N_4_	
	Initial	After Treatment	Initial	After Treatment	Initial	After Treatment	Initial	After Treatment
BOD_5_ (mg L^−1^)	12.7	9.0	7.0	4.5	6.2	3.0	13.3	8.5
COD (mg L^−1^)	52.0	23.0	12.0	6.5	34.0	6.3	47.0	21.0
BOD_5_/COD	0.24	0.39	0.58	0.69	0.18	0.48	0.28	0.40

## Data Availability

Data are contained within the article.

## Supplementary Materials

The following supporting information can be downloaded at: https://www.mdpi.com/article/10.3390/molecules28031170/s1, Table S1: Chemical structure, molecular weight, solubility in water (g L^−1^), pK_a_ and LogK_ow_ of pharmaceuticals detected in the WWTP secondary effluents.
