# Supplementary material for: Photocatalytic Degradation of Inherent Pharmaceutical Concentration Levels in Real Hospital WWTP Effluents Using g-C3N4 Catalyst on CPC Pilot Scale Reactor
Source: Molecules. 2023 Jan 25;28(3):1170. doi: 10.3390/molecules28031170 (PMC9919318; doi:10.3390/molecules28031170)
Supplement: Supplementary file 1 [file molecules-28-01170-s001.zip › molecules-2146217-supplementary.pdf]

SUPPLEMENTARY INFORMATION

Table S1. Chemical structure, molecular weight, solubility in water (gL^{-1}), pKa and LogKow of pharmaceuticals detected in the WWTP secondary effluents.

**Pharmaceutical**	**Chemical structure**[a]	**M.W.**	**Solubility in water (gL^{-1})**[a]	**pK_a**[a]	**LogK_{ow}**[b]
Amisulpride	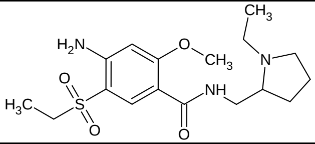	369.480	0.293	9.37	1.10
Atenolol	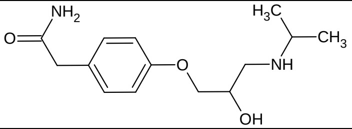	266.341	26.50	9.6	0.16
Carbamazepine	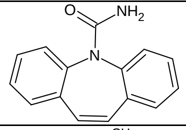	236.269	0.152	13.9 / 15.96[c]	2.45
Citalopram	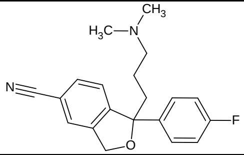	324.392	0.031	9.78	3.74
Mirtazapine	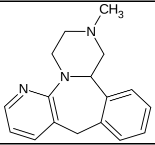	265.352	1.10	7.7	3.03
O-desmethyl venlafaxine	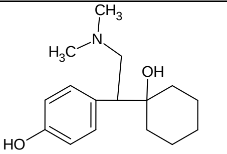	263.375	3.70	9.45 / 10.66	2.72
Quetiapine	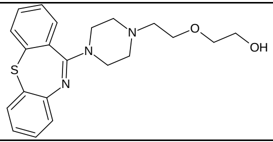	383.507	0.5869	2.78/7.46	1.94
Trimethoprim	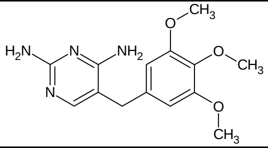	290.323	0.40	7.12	0.91
Valsartan	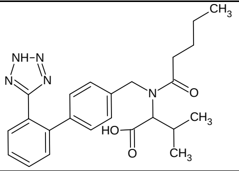	435.528	0.0014	3.6/4.73	4.00
Venlafaxine	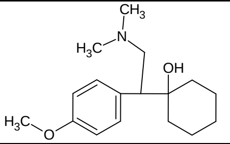	277.401	0.267	10.09	3.28

Literature data from: [a] Data provided by PubChem; [b] Data calculated by US Environmental Protection Agency's EPISuite™, available on ChemSpider; [c] Data calculated by ChemAxon, available on ChemSpider.
